# Knee isokinetic strength benchmarks in athletes across sports categories and performance levels

**DOI:** 10.5114/biolsport.2025.148534

**Published:** 2025-04-14

**Authors:** Raul Freire, Drake Huff, Brooke Butterick, Elias C. Figueroa, Jason C. Siegler

**Affiliations:** 1 Integrative Human Performance Lab, College of Heath Solution, Arizona State University, USA

**Keywords:** Muscle strength, Strength assessment, Muscle imbalance, Female athletes, Olympic sports

## Abstract

This study aimed to compare the isokinetic strength metrics (relative peak torque, hamstring/quadriceps (H/Q) ratio, and bilateral asymmetry) between male and female athletes and performance levels (National and International) across sports categories (Endurance, Power, Skill, Combat, and Team). Secondly, this study presents reference values for knee isokinetic strength assessments for athletes from different sports. This cross-sectional study evaluated the knee isokinetic peak torque (PT) at 60^o^ · s^−1^ in 355 athletes (182 women, age 24.2 ± 6.6 years, 173 men, age 24.3 ± 9.1 years) from 24 different Olympic sports. Two-way ANOVA and eta-square effect size were used to compare sex and performance levels across sports categories. Consistently higher mean PT values were observed for all isokinetic variables in males than in females (Extension: 15%, Flexion: 7%). Higher PT values were found among male and female athletes in Combat (Extension: 12%, Flexion: 18%, (p < 0.05) and Power (Extension: 11%, Flexion: 7%) (p < 0.05), respectively. No differences were found between performance levels for all isokinetic strength metrics (p = 0.10 to 0.98). H/Q ratio and bilateral asymmetry were not meaningfully affected by either sex or performance levels. In conclusion, male athletes demonstrated higher PT values than females, regardless of sports categories, suggesting PT benchmarks should be used across sports categories but not performance levels for male and female athletes.

## INTRODUCTION

Sex-related differences in strength have been widely investigated in diverse populations [1, 2]. In reference specifically to athletic populations [3–5], males produce more force than females, resulting from larger muscle cross-sectional area, longer limbs, muscle contraction speed, and fiber type [6, 7]. However, few studies have investigated sex-related differences in force production in well-trained or elite athletes, which may differ from lesser-trained individuals due to a more comparable training exposure [8, 9]. Moreover, studies have suggested that males and females present different strength development characteristics during puberty [10, 11]. Specifically, hamstring strength in females is lower than in males, often creating an imbalance between agonist and antagonist knee muscles. Indeed, females tend to use more of the quadriceps and less of the hamstring muscles to stabilize the knee and control the limb, which induces anterior shear stress to the tibia, subsequently increasing stress on the anterior cruciate ligament (ACL) and potentially contributing to a 4–6x greater risk of ACL injury in females [12].

The increased injury risk caused by strength differences and imbalances, in addition to the paucity of literature on this topic around elite female athletes, magnifies the importance of establishing normative values amongst high-level female athletes, with eventual aims of leveraging these data towards developing specific injury-prevention measures [13]. One relatively straightforward and easily implemented method to attain normative data is via isokinetic strength assessment. Isokinetic strength assessment has been used to help inform athletes’ injury rehabilitation/ prevention programs [14], assess muscular strength imbalances (bilateral or agonist-antagonist) [15], and identify deficiencies in muscular performance (e.g., maximal strength or power) in various sports [15]. Additionally, the conventional hamstring-to-quadriceps ratio (H/Q) attained from isokinetic assessment correlates with a greater incidence of lower limb injuries and thus has been used as a proxy for injury risk in sports, especially ACL injury [16–19].

Although the isokinetic assessment has been widely applied in sports settings, normative data for isokinetic strength profiles for well-trained or elite females for different sports and performance levels is scarce, making referencing or benchmarking difficult [20]. In practice, this information could be particularly useful during initial athlete evaluations. Clinicians, strength and conditioning coaches, and sports scientists working in elite sports would benefit from a comprehensive normative data set of knee isokinetic peak torque parameters to objectively compare with their peers across sex, type of sport, and level of performance classification. While some studies proposed exploring other isokinetic metrics beyond PT and H/Q [21], such as total work and power, there is a limited understanding of these variables compared to the research that includes PT and H/Q, especially in athletes. For example, little is known about the relationship between total work and power and sports performance metrics or injury risk, as we currently have for PT and H/Q [21]. Therefore, this study aimed to compare the isokinetic strength metrics (relative peak torque, H/Q, and bilateral asymmetry) between male and female athletes across sports categories (*Endurance, Power, Skill, Combat*, and *Team*) and performance levels (National and International). Secondly, this study presents reference values for knee isokinetic strength assessments for athletes from different sports.

## MATERIALS AND METHODS

### Experimental approach to the problem

A cross-sectional study design was implemented to investigate the sex-related differences in the knee isokinetic peak torque in a heterogeneous sample of athletes from different Olympic sports. Peak torque values from both limbs were compared between male and female athletes and categorized according to sports and performance level. The athletes visited the laboratory once for physical assessments in the morning, in which the isokinetic knee evaluation was part of a comprehensive physical evaluation (not included in this study). In the following order, athletes performed non-effort tests (e.g., anthropometry and resting metabolic rate) followed by vertical jump tests. The set of jumps consisted of three trials of each type of jump (squat and countermovement jump with arms fixed on the hip or free), totaling 12 jumps. Thirty seconds were allowed between each trial and 60 seconds between sets. The isokinetic test was performed last and at least 30 minutes after the vertical jump assessment.

### Subjects

Isokinetic testing was performed from 2017 to 2023 on 355 athletes (182 women, age 24.2 ± 6.6 years, 173 men, age 24.3 ± 9.1 years) from 24 different sports. Before testing, all athletes were given instructions for all testing procedures and made aware of potential risks before providing their written informed consent. All athletes were also cleared for participation by the resident medical officer and reported no lower limb injuries or pain on the test day. The typical H/Q ratio among healthy populations is between 50 and 80% [22]. Although athletes were cleared of injuries, tests that presented bilateral asymmetry of greater than 30% or a H/Q ratio lower or higher than 50% and 80%, respectively, were excluded from the analysis (48 athletes excluded, 21 women) [22]. These criteria were applied to avoid results from athletes in the rehabilitation process or chronic imbalances that could bias the sample. The study was approved by the city Health Council Research Ethics Committee (*blinded for review*) and conducted following the Declaration of Helsinki (revision of 2013).

### Procedures

Before the isokinetic assessment, athletes were weighed on a scale using standard athletic attire (women: shorts and top, men: shorts) (Welmy W200, Santa Bárbara d’Oeste, SP, Brazil). Subsequently, they completed a brief, standardized warm-up on a cycle ergometer (Wattbike Pro, Nottingham, UK) for five minutes at low to moderate intensity at 60–70 rpm (air gear lever on 2–4 and magnetic resistance lever on 0–2).

The isokinetic dynamometer Biodex System 4 PRO (Biodex Medical Systems, New York, NY, USA) was used to assess the knee’s extensor and flexor torque. Although our laboratory has not evaluated the test-retest reliability for this assessment, previous literature demonstrates a high level of reliability with this measure [23, 24] (ICC – 0.95–0.98). After the warm-up, athletes were seated in the chair, and the dynamometer was adjusted according to the participant’s characteristics. The athletes were secured on the chair with the help of soft straps on the upper body, hip, and thigh (evaluated side). The rotational axis of the knee (lateral femoral epicondyle) was aligned with the mechanical axis of the dynamometer, and the shin pad was fastened just above the lateral malleolus. The range of motion (ROM) was adjusted to the participant’s maximum flexion and extension knee angle, attempting to achieve at least 90° of ROM, considering 0° as a full extension. Gravity correction was applied at 30° knee flexion after directly measuring the lower limb lever arm system’s mass. A standardized set was performed before the test for warming up and familiarization with the procedures, which consisted of five submaximal concentric contractions performed at 60° · s^−1^ and five maximal repetitions (extension and flexion) at 60° · s^−1^ in the concentric-concentric mode. The highest torque achieved for knee extensor and flexor muscles was chosen for all analyses and expressed in relative (Nm/kg) units. The conventional H/Q ratio was calculated by dividing the highest hamstring peak torque by the highest quadriceps peak torque on dominant and non-dominant limbs for both sets. Finally, bilateral asymmetry was calculated as follows:

Bilateral asymmetry=((PT D-PT ND)/PT D)*100)

where PT D is the peak torque for the dominant leg, and PT ND is the peak torque for the non-dominant leg. Dominance was defined by asking which leg the athlete would use to kick a ball. The angular torque data was filtered for movement artifact noise using the proprietary software (Biodex Medical Systems). The software automatically identified the quadriceps and hamstring peak torque (and, consequently, the H/Q ratio) data. The peak contraction’s raw data (curve) was also visually inspected to ensure reliability.

### Statistical Analysis

The athletes were categorized by sex (males and females), performance levels [25] (National and International), and sports categories (*Endurance, Power, Skill, Combat*, and *Team*), adapted from Niebauer and colleagues [26]. Sports included in each category were determined as follows: *Endurance*: athletics (running events over 800 m and racewalking), sprint canoeing, sailing, triathlon, cycling, rowing; *Power*: athletics (field events, running events less than 800 m, and jumping events), golf, weightlifting, diving, surfing, badminton, tennis, table tennis, and swimming (50–200 m); *Skill*: skating, artistic gymnastic; *Combat*: fencing, taekwondo, karate, judo, and wrestling; *Team*: volleyball, beach volleyball, basketball.

Peak torque (PT) values, H/Q ratios, and bilateral differences were analyzed using a two-way ANOVA, followed by a Tukey post-hoc test with Bonferroni adjustment for multiple comparisons when appropriate. Partial eta squared (η^2^) was calculated to estimate the effect size (for ANOVA) and interpreted as “Small” (0.01–0.05), “Medium” (0.06–0.14), or “Large” (> 0.14). Hedge’s g was calculated to estimate the effect size of comparisons from sports categories, interpreted as “Small” (*g*: 0.2–0.5), “Medium” (*g*: 0.5–0.8), or “Large” (*g* > 0.8) [27]. In addition, Pearson’s correlation coefficient was calculated between bilateral difference (%) (extension and flexion) and isokinetic strength metrics (PT and H/Q) to investigate the relationship between them. The correlation strength was interpreted as *no relationship (r* < 0.20), *weak (r* – 0.20–39), *moderate (r –* 0.40–0.59), *strong (r* – 0.60–0.79), and *very strong (r* > 0.80). Percentile distributions were calculated for all isokinetic strength metrics [28], expressed in absolute (Nm) and relative (Nm/kg) units for both sexes and all sports categories. All data were presented as mean ± standard deviation, and the analysis was performed using the statistical package IBM SPSS version 29.0 (IBM Corp., Armonk, N.Y., USA), and the graphics were designed using GraphPad Prism (GraphPad Software, San Diego, CA, version 9.3.1). The significance level was set at *p* < 0.05 for all analyses.

## RESULTS

**FIG. 1:**
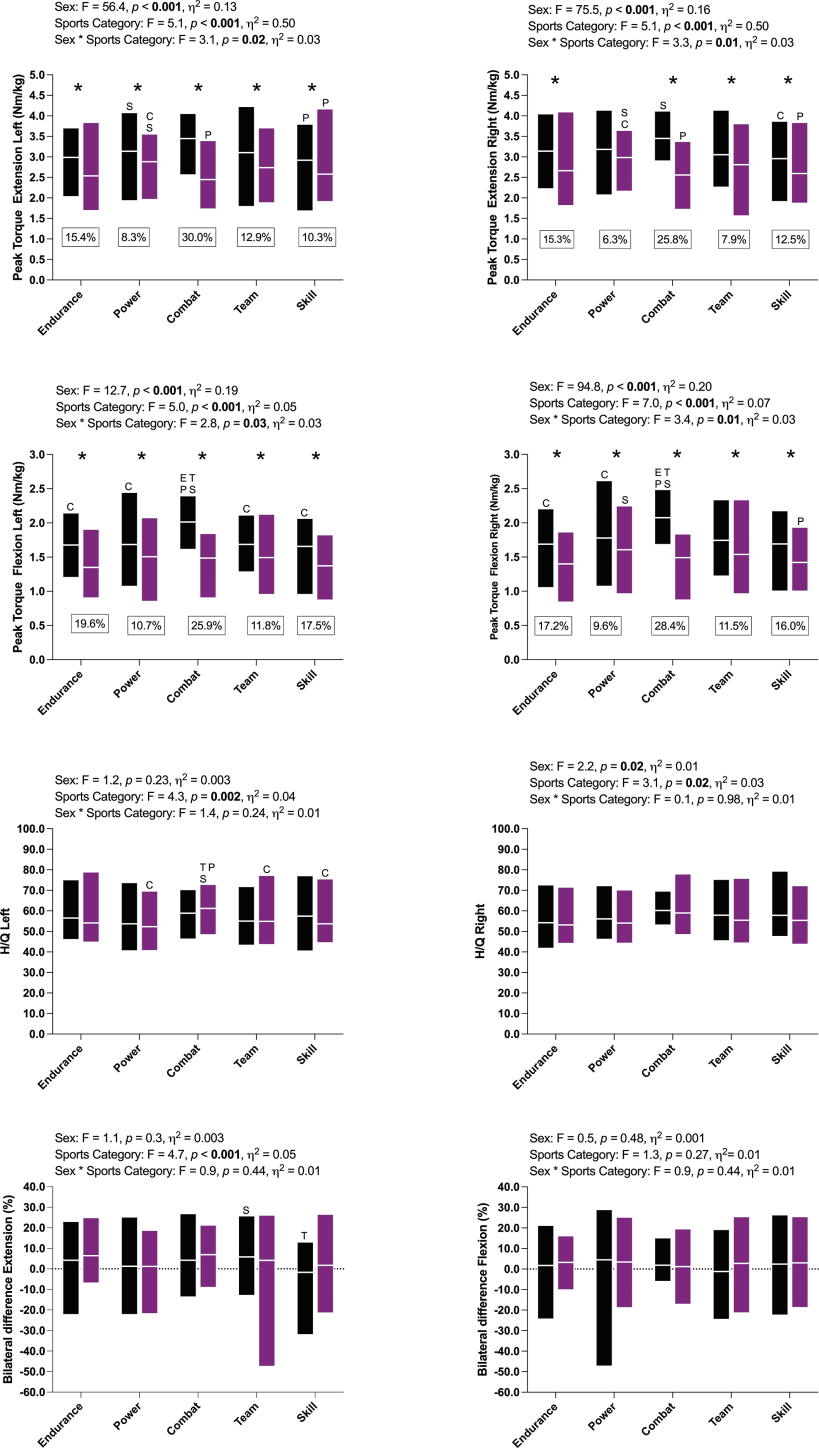
Data is presented as mean and min-max. Black bars: men, purple bars: women. *: statistical difference (p<0.05) between sex, letters mean statistical difference (p<0.05) across sports. E: Endurance, P: Power, C: Combat, T: Team, and S: Skill

Figure 1 presents the relative PT values of knee extensor and flexor muscles at 60º · s^−1^ between males and females across sports categories. Peak torque variables (extension and flexion) were significantly higher in males compared to females regardless of knee movement (extension and flexion), except PT Extension Right in Power athletes (*p* = 0.07; Figure 1). Hedge’s g calculations revealed medium to large (most large) effect sizes for all observed differences (*g*: 0.51–1.97). Combat athletes showed the greatest difference between sex regardless of knee movement and limb side (~25–30%), whereas Power athletes presented the lowest difference (~6–10%). Differences across sports categories were found in all PT variables. Combat athletes consistently presented higher mean PT values among males, whereas Power athletes were stronger among females. Hedge’s g calculations revealed medium to large (most large) effect sizes for all comparisons (*g*: 0.73–3.17). No bilateral differences between sex or sports categories (extension and flexion) were found. Mean bilateral differences were low (within ± 5%) for both sexes and sports categories. However, bilateral differences presented a large degree of variance (e.g., -50 to 25% in Team female and -50 to 30% in Power male athletes).

**FIG. 2:**
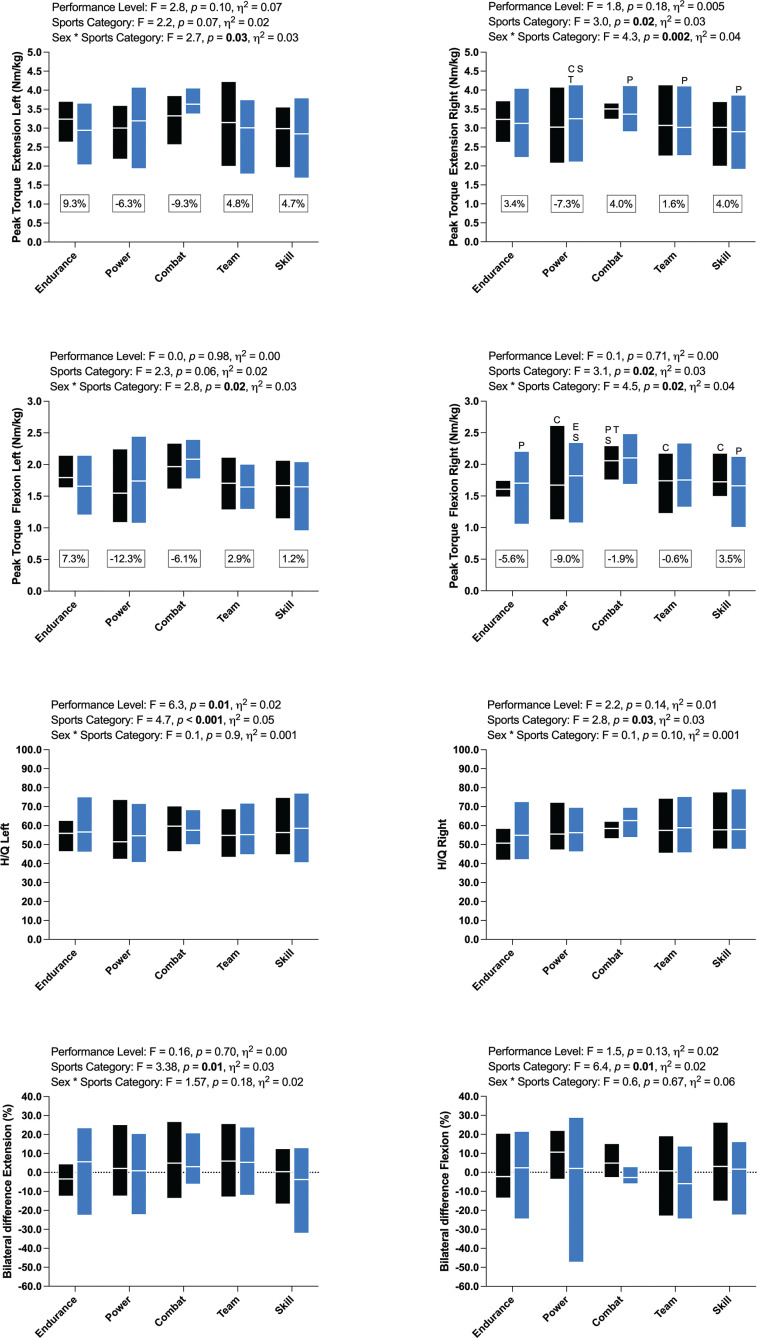
Data is presented as mean and min-max. Black bars: national, purple bars: international. *: statistical difference (p<0.05) between performance levels, letters mean statistical difference (p<0.05) across sports. E: Endurance, P: Power, C: Combat, T: Team, and S: Skill

Peak Torque variables were not statistically different between National and International performance levels, with average differences ranging between -0.6 and -12.3% (Figure 2). Differences across sports categories were found only for the right limb (extension and flexion). Combat athletes presented higher mean PT values compared to other sports categories in National and International athletes. Hedge’s *g* calculations revealed medium to large effect sizes (*g*: 0.59–1.24) among sports categories. H/Q ratios were similar across performance levels and sports categories (~50–60%), and mean bilateral differences were low (within ± 10%) for both performance levels and sports categories. However, similar to sexes and sports categories, bilateral differences presented a large degree of variance (e.g., -50 to 25% in Team females and -50 to 30% in Power male athletes).

Table S1 presents the correlation between bilateral difference and isokinetic strength metrics. At the most, weak (*r* < 0.40) correlations were found between these variables.

**Table 1:** Correlation between bilateral difference (%) and knee isokinetic strength metrics for male and female athletes.

	Males		Females	

	Bilateral difference (%)		Bilateral difference (%)	

	Extension	Flexion	Extension	Flexion
PT Extension Right (Nm · kg^−1^)	0.09* [-0.07–0.24]	0.04 [-0.12–0.19]	0.20* [0.05–0.34]	0.13 [-0.02–0.27]

PT Extension Left (Nm · kg^−1^)	-0.18 [-0.33– -0.02]	-0.30* [-0.44– -0.15]	-0.25* [-0.39– -0.10]	-0.04 [-0.19–0.11]

PT Flexion Right (Nm · kg^−1^)	0.05 [-0.11–0.20]	0.05 [-0.11–0.20]	0.03 -0.12– -0.18	0.26* [0.12–0.40]

PT Flexion Left (Nm · kg^−1^)	-0.18* [-0.33– -0.02]	-0.14 [-0.29–0.02]	-0.07 [-0.22–0.08]	-0.18 [-0.32– -0.06]

H/Q Right (%)	0.02* [-0.18–0.13]	0.05* [-0.11–0.20]	-0.22* [-0.36– -0.08]	0.15 [0.00–0.29]

H/Q Left (%)	-0.001 [-0.17–0.15]	0.19 [0.03–0.34]	0.23* [0.08–0.36]	-0.19 [-0.33– -0.05]

PT – peak torque, H/Q – hamstring to quadriceps ratio,

* – statistically significant correlation (*p <* 0.05)

## DISCUSSION

This study compared the isokinetic strength indexes (PT, H/Q ratio, and bilateral differences) between sex and performance levels across sports categories. This novel data set provides standard reference values for knee isokinetic peak torque (relative units, N · m · kg^−1^) for female and male athletes from different sports and performance levels to help practitioners with knee isokinetic test interpretation, notably when there are no previous records from the athlete. Males produced significantly higher PT values, regardless of knee exercise (extension and flexion) and limb side (right and left). These differences could have broader implications as to how normative performance values differ between males and females. Regarding sports categories, higher mean PT values for all isokinetic variables were observed in *Combat* (among males) and *Power* (among females) athletes. No meaningful differences in isokinetic strength between performance levels across sports categories were observed. H/Q ratios were also similar across sports categories and sex (around 60%), with no relevant effects on sports category or performance level.

When comparing across sex, several key measures contribute to the growing body of evidence surrounding elite female athletes. As previous literature has suggested, a 60% H/Q ratio has been considered optimal for reducing the risk of ACL injury [29–32]. Although injury incidence was not tracked in the present study, ACL injuries amongst female athletes remain one of the most trending sports-injury-related topics in women’s sports, as confirmed by Eldos et al. 2024 in a recent review [33]. Data presented here attempts to provide baseline isokinetic performance values for elite female athletes and support the value of 60% (Concentric-to-Concentric) at 60° · s^−1^, as seen in Table S1 to S5 (Supplementary Material), where the mean value is ~55–66% in females across both performance levels and sports categories. Although statistical differences were observed across sports categories on the left limb in the present study, we believe the differences do not represent clinical relevance. Further, the average (50^th^ percentile) relative flexion and extension peak torque values for both limbs for female athletes at 60° · s^−1^ were ~1.45 N · m · kg^−1^ and ~2.63 N · m · kg^−1^ for flexion and extension, respectively. Given the ACL injury frequency in female athletes and the utilization of the H/Q ratio to reduce injury, the present investigation provides further insight into isokinetic peak force values in healthy-elite female athletes across 24 sports.

To the extent of the authors’ knowledge, no study has comprehensively presented and compared knee isokinetic peak torque data across an extensive number of sports at different performance levels. Providing accessibility of data is fundamental for tests’ interpretation, particularly when the athletes evaluated have not previously been assessed with an isokinetic dynamometer. Most studies in the literature present isokinetic PT data comparing only a few sports (e.g., soccer, basketball, futsal, volleyball, handball, boxing, taekwondo, karate, and judo) [20, 34–37], whereas the present study profiled 24 separate Olympic sports organized in five sports categories.

França et al. [38] evaluated the knee isokinetic strength in 13 elite and 20 sub-elite athletes at the beginning of the season and five months later. The authors observed differences between groups only in relative PT flexion at the beginning of the season. Similarly, Sliwowski et al. [39] evaluated 100 Poland soccer athletes, 36 at the International level and at a similar age (~ 27 years old). Statistical differences between groups were evident only for flexion (in favor of the International level, 1.98 vs. 1.86 Nm/kg) [39]. The present study found no statistical differences between performance levels across all sports categories. The greatest mean difference (-12.3%, in favor of International) between National and International athletes was in PT Flexion on the left limb in Power athletes. Furthermore, the present data set suggests International Power athletes are consistently stronger than their National peers, whereas National Skill athletes are consistently stronger than their International peers (see Figure 2). A possible explanation for the divergent results might be homogeneity in the study sample across sports and sex. Sliwowski and colleagues [39] recruited a homogeneous sample of male soccer players, whereas the present study applied a pooled analysis including both sexes and several sports at the National and International levels.

Assessing and monitoring the strength and power levels of an athlete throughout the season and/or career is essential for ensuring safe return-to-play decisions, in addition to enabling precise training adjustments for optimal performance [40]. The present study provides mean values for knee isokinetic peak torque for different sport categories (expressed in absolute [Nm] and relative units [Nm/kg], see Supplementary Material Tables S2 to S6) with percentile distribution (P3^rd^ to P97^th^) for each sport category. This material can be used as an athlete-specific referencing benchmark for objectively interpreting isokinetic strength results. Representing the data across different sporting categories may broaden the application for practitioners working in underrepresented sports, even if not directly included in this analysis, thereby providing a theoretical baseline for sports that may have low numbers of players/athletes and no reliable or statistically robust normative values. Furthermore, percentiles can be used to compare the rank of an athlete in absolute and relative terms. For example, if an athlete ranks lower in relative but not absolute terms, one could speculate there may be a mismatch between strength and body composition or stature, or should body composition be appropriate, simply deficient in strength compared to their peers.

The present study has some limitations. Although many studies, particularly those focused on injury or the mechanism of injury, present dominant and non-dominant comparisons, this study did not. To reduce the potential for misusing the data, isokinetic peak torque values were presented for both limb sides and muscle groups, allowing the reader to draw their own interpretations. Despite the protocol including a familiarization set, we cannot objectively verify that all athletes gave a maximal effort. We also recognize that other metrics from the isokinetic assessment could be included (e.g., power, work, etc.), and some demographic variables were not included (e.g., training experience and injury history). Lastly, the isokinetic assessment was part of a comprehensive evaluation in which physical effort was performed before the isokinetic assessment. Ultimately, considering the crowded schedule of athletes for training, competition, and laboratory tests, we believe these limitations had a nominal impact on test performance. It is recommended that future research account for these limitations and begin to compare other performance assessments further to establish a more robust understanding of sex-related differences and, more importantly, to continue building normative performance values for elite female athletes.

## CONCLUSIONS

The present study demonstrates that male athletes are stronger than females in all isokinetic parameters, with the magnitude of difference being influenced by sport category. Sex, however, does not affect the H/Q ratio or bilateral asymmetry. Additionally, performance level does not affect any isokinetic strength parameter, including H/Q ratio and bilateral difference. Also, differences in isokinetic strength exist across sports categories regardless of sex and performance levels, with Power (among females) and Combat (among males) athletes being the largest in most analyses. Finally, this research provides reference values for knee isokinetic strength metrics in athletes, which could serve as a benchmark for practitioners working with this population.

## Supplementary Material

Knee isokinetic strength benchmarks in athletes across sports categories and performance levels
