# Supplementary material for: Knee isokinetic strength benchmarks in athletes across sports categories and performance levels
Source: Biol Sport. 2025 Apr 14;42(4):77–84. doi: 10.5114/biolsport.2025.148534 (PMC12492347; doi:10.5114/biolsport.2025.148534)
Supplement: Knee isokinetic strength benchmarks in athletes across sports categories and performance levels [file JBS-42-4-55764-s1.pdf]

Supplementary materials

TABLE S1. Normative data for knee isokinetic peak torque for extension and flexion in both sexes in *Endurance* athletes.

Percentile	Extension Left Nm [Nm/kg]		Extension Right Nm [Nm/kg]		Flexion Left Nm [Nm/kg]		Flexion Right Nm [Nm/kg]	
	Female	Male	Female	Male	Female	Male	Female	Male
97	216.3 [3.6]	286.6 [3.7]	229.6 [3.9]	309.4 [3.9]	113.2 [1.9]	174.8 [2.1]	107.9 [1.9]	173.4 [2.1]
95	206.9 [3.4]	285.1 [3.7]	218.9 [3.7]	305.7 [3.7]	112.2 [1.9]	173.0 [2.1]	106.8 [1.8]	167.7 [2.0]
90	195.4 [3.1]	282.8 [3.5]	207.2 [3.3]	300.4 [3.6]	100.6 [1.7]	162.3 [2.0]	103.8 [1.8]	155.5 [1.9]
85	189.6 [2.9]	280.5 [3.5]	201.4 [3.0]	291.5 [3.5]	89.7 [1.6]	152.9 [1.9]	101.0 [1.8]	148.5 [1.9]
80	183.1 [2.8]	276.3 [3.4]	192.5 [3.0]	281.4 [3.4]	89.2 [1.5]	144.3 [1.9]	99.2 [1.6]	146.1 [1.8]
75	179.0 [2.8]	267.3 [3.4]	186.5 [3.0]	269.2 [3.4]	88.5 [1.5]	136.9 [1.8]	94.7 [1.6]	143.9 [1.8]
70	175.2 [2.8]	253.0 [3.3]	180.0 [2.9]	251.1 [3.4]	88.0 [1.4]	134.0 [1.8]	92.2 [1.5]	136.7 [1.8]
65	171.8 [2.8]	247.6 [3.2]	173.0 [2.8]	249.2 [3.4]	87.3 [1.4]	133.7 [1.8]	90.8 [1.5]	125.9 [1.8]
60	168.7 [2.6]	240.9 [3.2]	165.5 [2.7]	239.3 [3.4]	86.2 [1.4]	130.1 [1.8]	89.4 [1.5]	124.7 [1.7]
55	165.0 [2.6]	237.7 [3.1]	163.8 [2.7]	235.3 [3.2]	85.2 [1.3]	123.5 [1.7]	88.6 [1.4]	122.7 [1.7]
50	155.0 [2.6]	234.0 [3.1]	162.6 [2.7]	231.1 [3.1]	83.8 [1.3]	119.5 [1.7]	87.5 [1.4]	121.3 [1.7]
45	145.2 [2.6]	228.1 [3.0]	161.5 [2.6]	226.6 [3.1]	82.4 [1.3]	118.3 [1.7]	86.1 [1.4]	120.7 [1.7]
40	142.3 [2.4]	221.5 [3.0]	160.1 [2.6]	223.5 [3.0]	81.4 [1.3]	117.9 [1.6]	84.4 [1.3]	119.2 [1.7]
35	138.6 [2.3]	216.0 [2.8]	149.8 [2.6]	219.1 [3.0]	81.4 [1.3]	117.6 [1.6]	82.9 [1.3]	113.5 [1.6]
30	134.2 [2.3]	206.9 [2.8]	142.3 [2.5]	218.7 [2.8]	79.8 [1.3]	113.9 [1.6]	81.5 [1.3]	110.6 [1.6]
25	129.5 [2.2]	200.9 [2.8]	133.8 [2.5]	213.1 [2.8]	75.8 [1.2]	111.9 [1.5]	78.3 [1.3]	110.3 [1.5]
20	124.6 [2.2]	190.9 [2.6]	120.5 [2.4]	207.1 [2.8]	68.8 [1.2]	106.2 [1.4]	71.6 [1.2]	107.9 [1.5]
15	116.7 [2.2]	182.4 [2.5]	118.1 [2.2]	201.3 [2.8]	66.9 [1.2]	102.4 [1.3]	67.0 [1.2]	105.5 [1.4]
10	110.5 [2.1]	174.0 [2.5]	116.1 [2.0]	194.8 [2.7]	63.9 [1.2]	99.4 [1.2]	63.8 [1.2]	104.2 [1.4]
5	103.6 [1.9]	162.5 [2.3]	111.2 [1.8]	184.4 [2.5]	58.7 [1.1]	92.9 [1.2]	58.3 [1.0]	102.7 [1.3]
3	99.5 [1.8]	161.2 [2.2]	106.8 [1.8]	175.9 [2.4]	55.2 [1.0]	92.2 [1.2]	53.5 [1.0]	101.0 [1.2]

TABLE S2. Normative data for knee isokinetic peak torque for extension and flexion in both sexes in *Power* athletes.

Percentile	Extension Left Nm [Nm/kg]		Extension Right Nm [Nm/kg]		Flexion Left Nm [Nm/kg]		Flexion Right Nm [Nm/kg]	
	Female	Male	Female	Male	Female	Male	Female	Male
97	268.5 [3.5]	358.7 [4.0]	262.2 [3.6]	398.2 [4.1]	151.8 [1.9]	203.8 [2.3]	166.7 [2.0]	247.5 [2.2]
95	251.5 [3.4]	331.4 [4.0]	260.7 [3.6]	393.9 [4.0]	132.8 [1.8]	191.1 [2.2]	137.6 [1.9]	224.6 [2.2]
90	238.8 [3.4]	312.8 [3.7]	235.9 [3.5]	333.0 [3.9]	122.8 [1.7]	175.4 [2.2]	126.5 [1.8]	170.2 [2.1]
85	229.3 [3.2]	295.8 [3.6]	229.0 [3.2]	311.8 [3.7]	114.0 [1.7]	166.5 [2.1]	118.0 [1.7]	167.5 [2.0]
80	212.6 [3.1]	282.0 [3.6]	220.1 [3.1]	287.8 [3.6]	102.7 [1.6]	160.7 [2.1]	112.8 [1.6]	162.0 [2.0]
75	207.8 [3.0]	269.9 [3.5]	207.8 [3.0]	282.8 [3.5]	100.2 [1.6]	151.3 [1.9]	105.5 [1.6]	155.2 [2.0]
70	198.7 [2.9]	263.3 [3.4]	190.6 [2.9]	280.8 [3.4]	97.8 [1.6]	146.6 [1.8]	97.9 [1.6]	151.9 [1.9]
65	184.7 [2.8]	251.9 [3.3]	184.1 [2.8]	265.0 [3.4]	97.1 [1.5]	142.8 [1.8]	97.4 [1.5]	144.4 [1.8]
60	179.3 [2.8]	247.4 [3.2]	170.8 [2.8]	257.7 [3.3]	93.5 [1.5]	135.1 [1.7]	91.3 [1.5]	138.4 [1.8]
55	170.1 [2.7]	239.7 [3.1]	164.5 [2.8]	243.7 [3.3]	89.4 [1.4]	132.8 [1.7]	89.8 [1.5]	135.1 [1.8]
50	164.9 [2.7]	236.0 [3.1]	162.2 [2.8]	235.3 [3.2]	83.8 [1.4]	127.4 [1.6]	88.0 [1.5]	132.9 [1.7]
45	157.9 [2.6]	232.9 [3.0]	154.8 [2.7]	230.4 [3.1]	78.6 [1.4]	122.4 [1.6]	85.6 [1.4]	132.2 [1.7]
40	143.4 [2.6]	229.5 [2.9]	150.2 [2.7]	226.7 [3.0]	77.7 [1.3]	120.1 [1.6]	81.9 [1.4]	123.5 [1.7]
35	139.8 [2.6]	219.6 [2.9]	148.2 [2.6]	218.6 [3.0]	74.4 [1.3]	117.1 [1.6]	78.6 [1.4]	121.1 [1.6]
30	138.5 [2.5]	211.0 [2.9]	138.6 [2.5]	212.2 [3.0]	72.4 [1.3]	111.8 [1.5]	77.6 [1.3]	117.1 [1.6]
25	135.7 [2.4]	205.3 [2.8]	130.9 [2.3]	207.3 [2.9]	69.9 [1.3]	107.2 [1.5]	76.3 [1.3]	113.3 [1.6]
20	132.1 [2.3]	199.3 [2.7]	125.8 [2.2]	196.2 [2.8]	66.8 [1.2]	103.2 [1.4]	69.4 [1.3]	108.5 [1.5]
15	120.2 [2.2]	191.8 [2.5]	123.1 [2.1]	186.8 [2.7]	60.7 [1.1]	98.3 [1.3]	66.6 [1.2]	106.0 [1.5]
10	110.6 [2.0]	173.7 [2.4]	120.0 [2.1]	178.7 [2.4]	59.9 [1.0]	94.7 [1.2]	65.7 [1.1]	100.5 [1.3]
5	99.3 [1.8]	163.5 [2.2]	111.6 [1.9]	168.1 [2.1]	52.3 [0.9]	90.3 [1.2]	59.7 [1.0]	87.9 [1.2]
3	93.8 [1.7]	155.9 [1.9]	107.3 [1.9]	164.2 [2.0]	51.0 [0.8]	87.1 [1.1]	52.7 [1.0]	87.2 [1.1]

TABLE S3. Normative data for knee isokinetic peak torque for extension and flexion in both sexes in *Combat* athletes.

Percentile	Extension Left Nm [Nm/kg]		Extension Right Nm [Nm/kg]		Flexion Left Nm [Nm/kg]		Flexion Right Nm [Nm/kg]	
	Female	Male	Female	Male	Female	Male	Female	Male
97	229.6 [3.2]	322.6 [4.0]	284.4 [3.3]	304.3 [4.0]	135.0 [1.8]	178.9 [2.4]	145.9 [1.8]	189.1 [2.5]
95	220.9 [3.1]	322.5 [4.0]	274.5 [3.2]	303.5 [4.0]	133.8 [1.8]	178.0 [2.4]	145.6 [1.8]	187.4 [2.4]
90	209.6 [2.9]	322.4 [3.9]	259.0 [3.1]	301.4 [3.8]	126.5 [1.7]	175.9 [2.4]	144.4 [1.8]	183.1 [2.3]
85	205.8 [2.8]	320.5 [3.8]	247.2 [3.1]	299.1 [3.7]	117.3 [1.7]	174.3 [2.4]	142.3 [1.8]	178.7 [2.3]
80	201.1 [2.7]	316.8 [3.8]	215.0 [3.1]	296.4 [3.7]	117.0 [1.7]	173.3 [2.4]	135.5 [1.7]	174.2 [2.3]
75	199.1 [2.7]	313.1 [3.7]	204.5 [3.1]	293.8 [3.7]	116.7 [1.6]	172.3 [2.3]	130.4 [1.7]	169.8 [2.3]
70	198.3 [2.6]	306.1 [3.7]	202.4 [3.0]	277.8 [3.7]	116.3 [1.6]	169.5 [2.3]	123.4 [1.7]	169.3 [2.3]
65	197.5 [2.6]	299.1 [3.7]	200.1 [2.9]	261.7 [3.7]	116.1 [1.6]	166.6 [2.3]	110.4 [1.7]	168.8 [2.3]
60	187.6 [2.6]	287.4 [3.6]	199.1 [2.8]	251.1 [3.6]	112.0 [1.5]	164.1 [2.3]	108.4 [1.6]	166.1 [2.3]
55	176.6 [2.5]	271.0 [3.5]	197.0 [2.7]	245.8 [3.4]	107.7 [1.5]	161.8 [2.2]	108.0 [1.5]	161.4 [2.3]
50	166.0 [2.5]	254.6 [3.4]	193.3 [2.5]	240.5 [3.3]	104.3 [1.5]	159.5 [2.2]	107.2 [1.5]	156.7 [2.3]
45	165.1 [2.5]	252.9 [3.4]	185.2 [2.5]	240.0 [3.3]	102.6 [1.5]	156.9 [2.0]	106.6 [1.5]	153.3 [2.1]
40	162.3 [2.4]	251.2 [3.4]	179.9 [2.4]	239.5 [3.3]	101.6 [1.5]	154.4 [1.9]	104.1 [1.5]	150.0 [2.0]
35	157.1 [2.4]	246.9 [3.3]	175.8 [2.4]	239.3 [3.3]	100.7 [1.5]	153.1 [1.8]	100.6 [1.5]	148.3 [1.9]
30	146.1 [2.3]	239.9 [3.3]	167.0 [2.2]	239.2 [3.2]	95.4 [1.4]	153.1 [1.8]	98.5 [1.4]	148.3 [1.8]
25	142.5 [2.2]	232.9 [3.3]	162.1 [2.1]	239.2 [3.2]	92.0 [1.4]	153.1 [1.8]	94.9 [1.4]	148.3 [1.8]
20	141.3 [2.1]	226.2 [3.3]	155.9 [2.1]	239.2 [3.2]	89.0 [1.3]	138.5 [1.7]	90.8 [1.4]	139.5 [1.8]
15	139.0 [1.9]	219.5 [3.3]	142.7 [2.1]	239.2 [3.2]	85.3 [1.1]	123.8 [1.7]	86.9 [1.2]	130.6 [1.7]
10	138.4 [1.8]	216.2 [3.3]	140.0 [2.0]	236.0 [3.1]	84.6 [1.0]	116.5 [1.7]	85.7 [1.0]	125.8 [1.7]
5	137.1 [1.5]	216.2 [3.3]	138.6 [1.9]	229.5 [3.0]	84.5 [0.9]	116.5 [1.6]	82.7 [0.9]	125.1 [1.7]
3	136.0 [1.2]	216.2 [3.2]	137.8 [1.8]	227.0 [2.9]	84.4 [0.8]	116.5 [1.6]	80.3 [0.9]	124.8 [1.7]

TABLE S4. Normative data for knee isokinetic peak torque for extension and flexion in both sexes in *Team* athletes.

Percentile	Extension Left Nm [Nm/kg]		Extension Right Nm [Nm/kg]		Flexion Left Nm [Nm/kg]		Flexion Right Nm [Nm/kg]	
	Female	Male	Female	Male	Female	Male	Female	Male
97	252.2 [3.6]	394.2 [3.8]	266.6 [3.6]	394.3 [4.0]	148.3 [2.0]	230.0 [2.1]	152.4 [2.0]	230.3 [2.2]
95	247.3 [3.5]	382.1 [3.8]	257.7 [3.6]	381.3 [3.8]	139.0 [1.9]	219.5 [2.1]	143.2 [1.9]	221.2 [2.1]
90	238.5 [3.4]	366.2 [3.7]	249.7 [3.5]	364.5 [3.6]	132.5 [1.8]	206.7 [2.0]	131.4 [1.9]	211.4 [2.1]
85	232.8 [3.3]	359.6 [3.6]	242.6 [3.4]	354.7 [3.5]	129.3 [1.8]	194.8 [1.9]	128.1 [1.8]	201.7 [2.0]
80	229.9 [3.2]	341.5 [3.5]	232.2 [3.3]	347.2 [3.5]	124.6 [1.8]	186.4 [1.9]	125.2 [1.7]	196.2 [2.0]
75	224.4 [3.1]	332.2 [3.5]	225.6 [3.2]	326.7 [3.4]	122.2 [1.7]	177.2 [1.9]	123.4 [1.7]	193.6 [1.9]
70	216.6 [3.0]	322.0 [3.3]	222.0 [3.1]	321.4 [3.3]	118.1 [1.6]	174.3 [1.8]	120.9 [1.7]	184.2 [1.9]
65	213.6 [2.9]	314.8 [3.3]	218.3 [3.0]	306.6 [3.2]	115.8 [1.6]	172.8 [1.8]	119.2 [1.6]	183.1 [1.8]
60	210.7 [2.8]	303.7 [3.2]	213.7 [2.9]	301.8 [3.2]	113.2 [1.5]	171.4 [1.7]	117.4 [1.6]	179.1 [1.8]
55	205.7 [2.8]	302.1 [3.1]	209.2 [2.9]	294.1 [3.1]	110.4 [1.5]	169.6 [1.7]	115.9 [1.6]	176.0 [1.8]
50	203.6 [2.7]	296.0 [3.1]	200.6 [2.8]	284.8 [3.0]	109.2 [1.4]	167.3 [1.7]	114.9 [1.5]	169.9 [1.8]
45	198.7 [2.7]	288.6 [3.0]	199.0 [2.8]	279.4 [2.9]	106.2 [1.4]	158.2 [1.7]	112.2 [1.5]	161.4 [1.7]
40	193.8 [2.6]	281.0 [3.0]	196.6 [2.6]	267.0 [2.9]	102.5 [1.4]	157.2 [1.6]	108.2 [1.5]	158.3 [1.7]
35	187.4 [2.5]	272.2 [3.0]	193.6 [2.6]	260.2 [2.7]	100.6 [1.4]	150.1 [1.6]	107.2 [1.4]	153.2 [1.6]
30	180.8 [2.4]	269.8 [2.9]	191.3 [2.5]	251.1 [2.7]	99.2 [1.3]	146.8 [1.6]	105.7 [1.4]	146.8 [1.6]
25	178.8 [2.4]	262.9 [2.8]	183.9 [2.5]	241.8 [2.6]	97.8 [1.3]	139.2 [1.5]	102.4 [1.4]	144.0 [1.6]
20	165.1 [2.3]	257.4 [2.6]	177.3 [2.3]	236.5 [2.4]	96.8 [1.3]	135.1 [1.5]	96.3 [1.3]	137.1 [1.5]
15	158.2 [2.1]	243.7 [2.6]	168.9 [2.3]	219.0 [2.3]	93.5 [1.2]	132.0 [1.4]	94.2 [1.3]	131.9 [1.5]
10	150.1 [2.0]	228.7 [2.5]	155.5 [2.1]	195.2 [2.2]	89.6 [1.2]	130.2 [1.4]	89.0 [1.2]	124.6 [1.4]
5	138.2 [1.9]	197.2 [2.0]	127.5 [1.8]	188.6 [2.1]	86.0 [1.1]	125.0 [1.4]	80.8 [1.2]	121.3 [1.3]

TABLE S5. Normative data for knee isokinetic peak torque for extension and flexion in both sexes in *Skill* athletes.

Percentile	Extension Left Nm [Nm/kg]		Extension Right Nm [Nm/kg]		Flexion Left Nm [Nm/kg]		Flexion Right Nm [Nm/kg]	
	Female	Male	Female	Male	Female	Male	Female	Male
97	183.3 [3.3]	238.3 [3.4]	180.2 [3.3]	235.6 [3.5]	93.7 [1.7]	142.7 [2.0]	95.0 [1.7]	137.7 [2.0]
95	179.1 [3.2]	235.3 [3.4]	178.2 [3.3]	231.5 [3.4]	91.0 [1.7]	139.1 [2.0]	94.1 [1.7]	134.4 [2.0]
90	164.0 [3.0]	226.7 [3.3]	173.0 [3.1]	227.2 [3.3]	89.6 [1.6]	128.9 [1.9]	90.4 [1.7]	134.2 [2.0]
85	162.8 [2.9]	226.0 [3.3]	165.9 [3.0]	226.4 [3.2]	86.6 [1.6]	123.1 [1.9]	89.2 [1.6]	132.6 [1.9]
80	158.8 [2.8]	224.2 [3.3]	162.1 [2.9]	221.5 [3.1]	85.1 [1.6]	119.6 [1.9]	86.3 [1.6]	130.3 [1.8]
75	157.6 [2.7]	214.2 [3.1]	158.3 [2.9]	219.2 [3.0]	83.6 [1.6]	118.8 [1.8]	85.3 [1.6]	124.1 [1.8]
70	154.4 [2.7]	203.3 [2.9]	156.9 [2.9]	209.7 [3.0]	82.8 [1.5]	117.6 [1.7]	82.3 [1.5]	118.6 [1.8]
65	143.9 [2.7]	201.5 [2.9]	149.4 [2.8]	204.4 [2.9]	80.3 [1.5]	116.9 [1.7]	77.3 [1.5]	116.9 [1.7]
60	136.9 [2.6]	197.5 [2.9]	141.5 [2.7]	200.9 [2.9]	78.2 [1.4]	116.2 [1.7]	76.7 [1.5]	116.2 [1.7]
55	133.4 [2.6]	195.1 [2.8]	136.6 [2.6]	195.9 [2.8]	76.9 [1.4]	115.8 [1.7]	76.1 [1.5]	113.4 [1.7]
50	128.2 [2.5]	175.9 [2.7]	129.5 [2.5]	191.4 [2.8]	75.1 [1.4]	113.2 [1.6]	75.7 [1.5]	108.7 [1.7]
45	121.9 [2.5]	175.6 [2.7]	125.4 [2.4]	183.1 [2.7]	72.0 [1.4]	110.2 [1.6]	75.4 [1.5]	107.6 [1.7]
40	120.3 [2.4]	173.5 [2.7]	121.6 [2.4]	180.3 [2.7]	69.4 [1.4]	106.9 [1.6]	74.6 [1.5]	105.1 [1.6]
35	119.0 [2.4]	160.3 [2.6]	117.8 [2.3]	171.9 [2.7]	68.5 [1.3]	105.6 [1.6]	71.9 [1.4]	102.1 [1.5]
30	115.2 [2.3]	149.1 [2.4]	114.3 [2.3]	164.4 [2.7]	67.9 [1.3]	102.0 [1.6]	69.2 [1.3]	99.1 [1.5]
25	111.7 [2.3]	144.6 [2.1]	108.0 [2.3]	162.2 [2.6]	66.7 [1.3]	101.2 [1.5]	68.4 [1.3]	96.9 [1.5]
20	109.1 [2.3]	139.9 [2.1]	105.9 [2.2]	156.4 [2.5]	61.6 [1.3]	97.9 [1.5]	66.3 [1.3]	94.0 [1.5]
15	108.9 [2.2]	139.1 [2.0]	104.3 [2.1]	148.6 [2.4]	56.4 [1.2]	93.5 [1.5]	61.5 [1.3]	92.3 [1.4]
10	106.0 [2.2]	136.6 [1.9]	100.0 [2.0]	127.3 [2.0]	54.2 [1.2]	91.8 [1.4]	56.5 [1.2]	88.7 [1.3]
5	88.7 [2.1]	120.8 [1.9]	85.4 [2.0]	85.3 [1.3]	44.5 [1.1]	89.6 [1.3]	46.4 [1.2]	86.1 [1.3]

Articles published in the Biology of Sport are licensed under an open access Creative Commons CC BY 4.0 license.
